# Gelseminum Sempervirens, as a Remedy in Gonorrhœa

**Published:** 1861-07

**Authors:** T. A. Means

**Affiliations:** Memphis, Tennesse


Medical and Surgical Journal.

Vol. VI.] JULY, 1861. [No. 11

### ORIGINAL COMMUNICATIONS.

### ARTICLE I.

*Gelseminum Sempervirens, as a remedy in Gonorrhœa.* By T. A. Means, M. D., of Memphis, Tennessee.

The disease which I am about to consider, although common to every class and condition of the adult race, has claims of no ordinary character, and should merit special attention from the physician as well as medical phylosopher. Its extent and frequency has lead to every experiment—and experiment to empiricism—and while each one assumes the responsibility ofphysician, arrests the affection *suo marte*, and then tenders the knowledge of his experience to his friend and fellow. By this general and gratuitous dissemination of experimental knowledge, the physician has been robbed, in a measure, of a disease legitimately within his province, at least its chronic stage, or sequela, are alone left him to combat.

In the acute or primary stage of the affection, the service of a physician is seldom asked for, his advice unsolicited, and not until experiment has exhausted itself, and the disorder become less tractable, is he favored with full control of the case. The uninformed, then, create a secondary affection out of a primary, whilst the physician treats only the obstinate, or continued symptoms. This is much to be regretted, and I fear the physician is at fault, to a considerable extent, by willingly, and much too readily, yielding up cases presented them to those who cannot adequately appreciate the nature or extent of the disease with which they are affected, nor the sympathetic and secondary disorders, which are sure to follow the injudicious, as well as indiscrimate use of remedies.

I might enlarge on this subject to the extent justified by its importance, were I so inclined, but I propose only to record my experience in the treatment of Gonorrhœa, with Gelsemin, an article heretofore considered of little therapeutic value, in the treatment of this affection. I have ventured, therefore, to give, somewhat in detail, the temperament, appearance, etc., of each, less to extend this article, than to show its effect upon constitutional peculiarities.

A gentleman, aged 18 years, of good address, came to my office in the latter part of September last, with Gonorrhœa of one week's standing, which he had aggravated rather than controlled, by the use of so-called infallibles, gathered from books, journals, and the uninformed. Complete confidence in his own skill, with the belief that his case was simple and readily arrested, led to the indiscriminate use of balsams, and astringent injections, disregarding altogether the neceessary hygiene. Then, impelled by fear, lest his disorder should assume a more aggravated form, and thereby prevent his marriage, (then but a fortnight off,) sought my advice, with the following appearance and condition.

Stature, medium; temperament sanguine; voice weak; look, wild and suspicious. The animal was plainly visible, notwithstanding his address and apparent manhood. He said, that freedom from parental restraint led him into vice and unrestrained venal excess; and that he totally disregarded the choice of material, until entrapped. The disease manifested itself on the second day, and, on the eighth of infection, I was consulted, and prescribed, as an experiment, the *Gelseminum sempervirens*, recommended to the Profession first by Dr. John Douglass, of South Carolina, as a remedy in Gonorrhœa—then by Professor Procter, of Philadelphia—by Dr. Johnson of this State, and subsequently by others, prominent and responsible.

I ordered a saturated tincture of the bark of the root, a teaspoonful in a wine-glass of water, to be taken three times daily, fifteen minutes before each meal. To abstain from all oleaginous food, wines or malt liquors, and to report to me the moment he experienced a dimness of vision, which I assured him would result, and therefore not give himself any alarm, as it would soon pass off, and with it, his disorder. I requested that he should visit me once daily, always before meals, and whilst under the influence of the medicine. He appeared on the following morning, stating that he experienced no unpleasant effects from the first dose, but that the second produced, within one hour, such a clouded vision, that he could scarce distinguish the most prominent colors. In this condition I saw him, yet could discover nothing in the appearance of his eyes, or features, other than natural. He complained of an unpleasant "haze," accompanied with dryness of the ball, which induced him to wink much oftener than natural. This annoyance passed off within three hours, yet with no perceptible alteration in the nature or extent of the disease. I requested him to continue the use of the tincture as before; to keep his mind as far as possible from his malady, and to report to me as requested.

On the third morning I found the urethra less sensitive, the discharge thin and scant, and the effect of the Gelsemen less decided. On the fourth day I was pleased to find my patient free from inconvenience or pain. On the fifth day, morning, I discharged him, with the advice, to abstain from all animal excitement, and live strictly temperate for at least ten days.

CASE II.—Mr. B., decidedly plethoric, brown face, features round and full; voice husky and feeble; had contracted the disease a fortnight before, and believed himself badly treated by a physician in the outset, who advised the repeated application of caustics, which greatly aggravated the discharge, and rendered his suffering intolerable.

He told me his breakfast consisted of capsules, and that a strong solution of zinc, catechu and opium was thrown up the urethra three times daily, followed each time by painful erections. The meatus was much inflamed and swolen. The canal pained him its whole extent; priapism was almost constant with its attendant chordee, whilst the dread of rupture kept him restless and awake. Cold cloths were constantly applied to the glans penis, over the pubis, and to the perenium. Four nights passed in this way, when, on the fifth day, I prescribed the Gelsemen, as before, only in smaller quantities, as the sensibility and tumefaction of the walls of the urethra, with his full habit, and a superabundance of the adipose, did not seem to justify, in the outset, so large a dose as in Casse I. On the following morning he came to me much frightened, and fully under the influence of the medicine. I requested him to remain in-doors, and report to me the moment sight was restored. He stated, that within three hours, all external objects appeared natural. That he could micturate without pain, and felt encouraged to continue the use of the Gelsemen, as before. I saw him on the following evening, when he reported freedom from erections; some little soreness along the canal; natural and refreshing sleep; scant discharge, and disappearance of all other unpleasant symptoms. Discharged, cured.

Case III.—Mr. T., of opposite build, full six feet in height; temperament highly nervous; hair sandy, approaching red; was a boat-hand by trade, and exposed much to night air. He appeared much worn by almost constant dissipation, and had suffered from four attacks of Gonorrhœa, each increasing in severity. His whole physique told too plainly the dissolute life he had led; yet his condition did not seem to concern him much, although the discharge from the urethra was immoderate. Priapism became instant and complete, from the slightest touch or warmth, and, so sensitive was the glans and meatus, that he was forced to carry his hand in his breeches pocket, to support and keep at ease his genitals. He had two sympathetic bubos; and intolerant pain in the left poplitial space. His nights were consumed in constant watchfulness, and repeated application of cold cloths, and douches, to prevent erections.

Mr. T. had been taking, as the two preceeding, the old stereotyped remedies, balsams, zinc, etc. He begged me, to suspend the use of injections, and said he was willing to put up with the nauseating balsam, for while the former was a friend to his stomach, the latter was an enemy to his urethra. I again administered the Gelsemen in full doses, and ordered him to empty his bladder before retiring, and to apply a wet bandage, quite tight, the entire length of the organ. This seemed to stay the erections, and control, to some extent, the discharge.

His sight was not readily affected, but was, perhaps, more obscure when fully under the influence of the medicine. On the fourth day, all the more prominent symptoms had disappeared, and nothing remained but a slight discharge, which I readily corrected by a few injections of thin mucilage of Port Wine and Acacia. Discharged, cured.

The profession, junior, are, perhaps, too tardy in accepting a remedy pronounced new, until sanctioned by older and wiser heads; and I must own myself embarrassed in testing the virtue of the Gelsemen, until I had known of its effects, yet, the repeated and ineffectual attempts to arrest the disease, by the ordinary oleo-resins, astringents, etc., etc., led me to accept any remedy, whether novel or accredited, that would supplant their use.

The medicinal properties of this plant, have been long known, and used with marked success, in the treatment of Rheumatism and arthritic affections. Dr. B. Keith, of New York, has used the Gelsemen successfully, in controlling the pulse in fevers; in hemorrhage from the lungs, bowels, uterus, and urinary organs; in dysentery, spermatorrhœa, amaurosis, catarrh, and dry cough dependent upon irritation of the throat, in doses of half a grain, three times daily. In profuse uterine hemorrhage, he considers it quite a specific, and adds, that, in dysentery and bowel affections, from one-tenth to one-eighth of a grain, administered after each discharge, will speedily arrest all hemorrhage and traces of the disease.

The discovery of the medicinal properties of this plant, was accidental. "A planter of Mississippi laboring under an obstinate bilious fever, directed his servant to get a particular root from his garden, and prepare a tea from it. The tea was prepared accordingly, and drank by the invalid, who was soon after affected with great prostration, and especially muscular debility, so that he could not raise a limb, but without stupor. These effects gradually disappeared, and with them the fever. The servant had made a mistake in the root, and dug that of the Gelseminum, instead of the one intended. The planter having made this discovery, employed the root afterwards, with success, in all cases of fever, especially intermittent.

It eventually fell into the hands of professional quacks, and was placarded as the "Electric Febrifuge." It seems to have been unknown, however, as a therapeutic agent in the treatment of Gonorrhœa, until called to the notice of the Profession by Dr. —— Douglass, of ———————— who furnished a short note of its value to the *Charleston Medical Journal*, 1857, stating that he had employed it, at the instance of one of his pupils, some thirty years before.

The Gelseminum is said to be febrifuge, dependent upon its relaxing and antispasmodic properties; is used in all types and grades of fever, save the conjestive; and when combined with Quinine, in the proportion of eight drops of the tincture to three grains of the salt, given every two or three hours, proves invaluable in controlling the pulse in typhus and typhoid fevers. Its effects are decidedly narcotic, producing clouded vision, double-sightedness, partial or complete muscular prostration, and inability to open the eyes with freedom, all of which soon pass off, leaving the system uninjured, and free from fever.

My experience with this remedy, is yet, of course limited, and I could not therefore recommend its use, uncombined, in all stages of Blenorrhagia, with the assurance of immediate and permanent relief. I am convinced, however, that in it we have a powerful agent, when used alone, in controlling the disease in its inflammatory stage, and when combined with Balsam Copavia, or Fluid Extract of Buchu, proves highly beneficial in the advanced or chronic stage. On the score of humanity, therefore, it should at least have an impartial trial. If there exists stricture of the urethra, chronic affections of the prostrate gland, affections of the seminal ducts, hemorrhage, etc., etc., or some of the accidents—such as Cystitis, Nephritis, and Orchitis, then we should be cautious in the outset, when administering it alone. These latter affections, or consequences, are obstinate and sometimes very severe, requiring at once the strictest antiphlogistic means and hygienic rules.

The formula which I have been in the habit of using, consists of equal parts of Tinct. Gleseminum, Fluid Extract of Buchu or Balsam Copavia and Water. The Buchu I much prefer, as it is more palatable, less offensive to the patient, and entirely free from that excessive nausea which so often attends the use of the oleo-resin. It is especially indicated, when there exists any morbid irritation of the urethra, retention, or incontinency of urine, resulting from atony of the parts concerned in its evacuation; and, when combined with Gelsemin, in the proportions mentioned on the preceeding page, proves highly beneficial in controling that discharge, usually called a *gleet*, or chronic inflammation of the urethra, with or without analogous inflammation of its appendages. I do not intend to prescribe rules of treatment adapted to every constitution and diathesis, as these must be met by auxiliary means and measures. My only purpose in writing this essay, was to introduce a remedy heretofore but little known to the profession, in the treatment of Blenorrhagia. I therefore submit the foregoing facts, confidently believing, if the remedy is judiciously administered, with a strict observance of hygiene, we may be freed from those innumerable visits of complaint and injury, which falls to the lot of every physician having a case in hand.

I am unable to explain satisfactorily, the *modus operandi* of this article. Its effects are, in some respects, similar to those of Copavia; it diminishes the force and frequency of the pulse, and by being taken into circulation, reaches the kidneys, imparting to the urine certain medical properties, which, upon its passage out, produces a beneficial effect upon the living membrane of the urethra.

